# Bilateral Progressive Idiopathic Annular Lipid Keratopathy

**DOI:** 10.1155/2012/731413

**Published:** 2012-05-10

**Authors:** Ramon C. Ghanem, Vinícius C. Ghanem, Gustavo Victor, Milton Ruiz Alves

**Affiliations:** ^1^Department of Cornea and Refractive Surgery, Sadalla Amin Ghanem Eye Hospital, Joinville, SC 89201, Brazil; ^2^Department of Ophthalmology, University of São Paulo, São Paulo, SP 05403-000, Brazil

## Abstract

*Purpose*. To report two unusual cases of idiopathic lipid keratopathy with symmetrical bilateral annular corneal lipid infiltration and describe confocal microscopy findings. *Methods*. Case reports. *Results*. We report two patients with bilateral peripheral deep stromal lipid deposits beginning in an arcuate pattern and progressing to a complete annular shape. Cholesterol crystals were observed in the paracentral area in both cases with characteristic crystalline-like structures in the confocal microscopy. Deep thin corneal blood vessels were observed in one patient, but no cause for then was established, despite decades of followup. This patient had an idiopathic limbitis as well, occurring in episodes. No previous ocular trauma, systemic disease or family history was reported for both cases. *Conclusion*. These two cases of idiopathic annular lipid keratopathy were observed for more than a decade with documented slow and insidious progression of the infiltrates, in spite of the use of topical steroids in one case. In the majority of other reported cases, a penetrating keratoplasty was made necessary. Differently, we showed that the visual acuity can remain quite good for years with very slow deterioration.

## 1. Introduction

 Opacification of the cornea by lipid deposits may be primary (with no evidence of prior corneal inflammation or vascularization) or secondary (resulting from systemic or local disease). Secondary lipid keratopathy associated with previous ocular injury or disease, which results in corneal vascularization, is not uncommon. It is often an unilateral and localized disease with a known cause [1]. The purpose of this paper is to report two unusual cases of idiopathic lipid keratopathy with symmetrical bilateral annular (ring-like) corneal lipid infiltration and describe their confocal microscopy findings.

## 2. Case Reports

**Patient 1**

**Figure 1:**
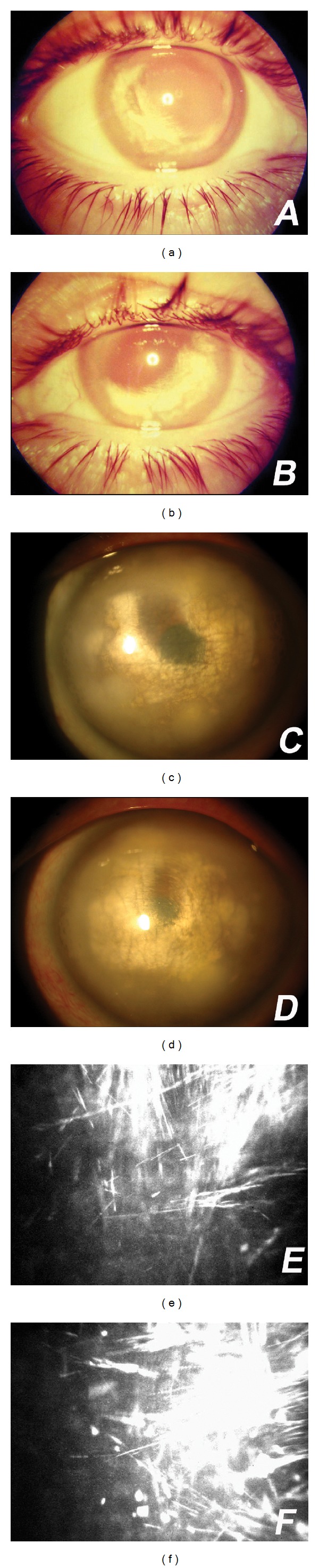
(a and b) Inferior white corneal infiltrates bilaterally in a crescent shape inferotemporally. (c and d) Progression to a complete bilateral annular lipid infiltration in the stroma of both eyes with cholesterol crystals in the paracentral area. (e and f) Confocal microscopy revealed crystalline structures in the stroma bilaterally.

A 39-year-old white woman had a history of white spots on both corneas, beginning in the inferotemporal peripheries, since 1985. She noticed progressive peripheral opacification of both corneas in an arcuate pattern and in 1994 sought an ophthalmologist. Examination showed inferior white infiltrates in both eyes in a crescent shape inferotemporally (Figures 1(a) and 1(b)). No previous ocular trauma or systemic disease was reported, but she had a history of sporadic asymptomatic red eye. There was no family history of eye disease. In 2004, uncorrected visual acuity was 20/25 in the right eye and 20/20 in the left. Slit-lamp examination revealed complete bilateral annular lipid infiltration in the medium and deep stroma of both eyes with cholesterol crystals in the paracentral area. A central area of the cornea of about 2 mm in the right eye and 1 mm in the left were clear of cholesterol crystals but showed a faint haze in both eyes (Figures 1(c) and 1(d)). Confocal microscopy revealed highly reflective crystalline structures in the anterior and middle stroma of both eyes (Figures 1(e) and 1(f)). No corneal blood vessels or other ocular abnormalities were observed. Systemic evaluation was unremarkable. Laboratory determination of the blood lipidogram was normal. In the last 5 years, she remained asymptomatic without medication and maintained stable visual acuity.

**Patient 2**

**Figure 2:**
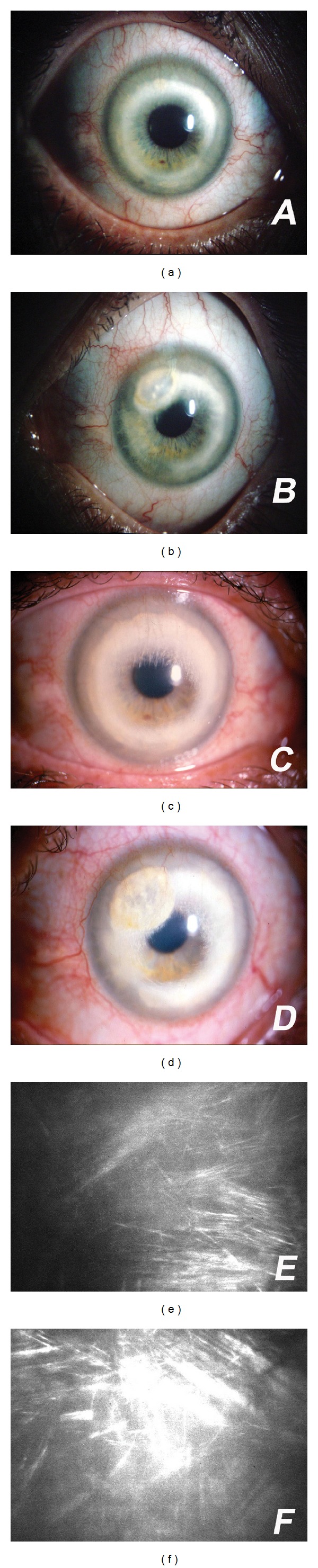
(a and b) Annular lipid infiltration in the corneal stroma of both eyes with cholesterol crystals in the paracentral area and deep corneal blood vessels from the limbus to the deposits. (c and d) Progression of the infiltration in both eyes, threatening the visual axis. (e and f) Confocal microscopy revealed crystalline structures in the stroma bilaterally.

A 42-year-old white woman had a slowly progressive yellow-white peripheral annular corneal deposit on both corneas since 1980. She complained of a progressive centripetal opacification over a 20-year period associated with a history of red eyes, ocular irritation, and photophobia, occurring in crisis, 3 to 4 times a month, worse in the left eye. No previous trauma or systemic diseases were reported. There was no family history of eye disease. Examination during these episodes showed conjunctival hyperemia, dilated limbic and corneal blood vessels, suggestive of an idiopathic low-to-moderate limbitis. No anterior chamber reaction, corneal edema, or lid disease was observed. She has been followed in our institution for more than a decade with the diagnosis of bilateral lipid keratopathy secondary to an idiopathic limbitis. During this period, a 6-month treatment with topical cyclosporine 2% four times a day instead of topical steroids was attempted. Without control of the inflammation and complains of ocular burning, the medication was stopped. For the last 5 years, she is on topical fluorometholone 0.01% or loteprednol 0.2%, 1 to 3 times a day, with relative control of the inflammation, no intraocular pressure rise, but considerable progression of the corneal deposits was observed. In 1999, examination showed annular lipid infiltration in the medium and deep stroma of both eyes with cholesterol crystals in the paracentral area (most severe superiorly) and deep corneal blood vessels from the limbus to the deposits. In the left eye a dense superotemporal elliptical lipid infiltration, separated from the limbus by 1 mm of clear stroma, could be seen as well. The central cornea area of about 6 mm was clear in both eyes (Figures 2(a) and 2(b)). At present, slit-lamp examination reveals progression of the infiltration in both eyes, threatening the visual axis (Figures 2(c) and 2(d)). Best spectacle-corrected visual acuity (BSCVA) dropped in a 10-year period only in the left eye from 20/20 to 20/30. In the right eye, the BSCVA remains 20/20. Cycloplegic refraction is −3,75 D in the right eye and −3,25 D in the left. Confocal microscopy revealed crystalline-like structures in the stroma bilaterally (Figures 2(e) and 2(f)). No other ocular abnormalities were observed. Extensive systemic evaluation was performed twice and was unremarkable. Laboratory determination of the blood lipidogram was normal.

## 3. Discussion

The primary form of lipid keratopathy is based on the presence of lipid deposits in the cornea with no prior vascularization or inflammatory alteration [2], and in the absence of serum lipid elevation. It is usually bilateral and may decrease vision in advanced cases. The accumulation of lipids in these cases may be due to excessive lipid production or deficient capacity for lipid metabolization [3], possibly an exaggeration of the same process that produces an *arcus senilis* [2]. One of our cases showed no corneal vascularization and may be called, primary and the other showed peripheral corneal neovascularization and some form of local inflammatory process, so by definition classified as secondary lipid keratopathy.

The secondary lipid keratopathy is related to the presence of corneal blood vessels, as described by Cogan and Kuwabara [1]. The typical presentation is a dense, yellow-white infiltrate in the corneal stroma around an area of vessels. Corneal lipid deposits are usually unilateral, localized, and found after ulceration, trauma, hydrops, interstitial keratitis, or herpetic keratitis [1, 4]. Other causes of bilateral centripetal lipid keratopathy include diffuse anterior scleritis [5] and Cogan's Syndrome [6]. Occasionally, lipids may be deposited in the cornea secondary to systemic dyslipoproteinemias, including lecithin-cholesterol acyltransferase deficiency, Tangier disease (familial high-density lipoprotein deficiencies) and Fish-eye disease [7]. In lipoprotein disorders, the involvement is usually bilateral without corneal vascularization and associated with abnormal serum lipoproteins.

Some cases of secondary lipid keratopathy have been termed idiopathic because they present corneal neovascularization of unknown cause. Most previous reports described localized corneal deposits with underlying neovascularization [8, 9]. In 1991, Durán and Rodriguez-Ares reported the first two cases, to our knowledge, of idiopathic annular lipid keratopathy [10]. Our case 2 is similar to those in its bilateral symmetrical presentation with crescent shape, annular deposits, and deep stromal vascularization from the limbus in the direction of the corneal deposits. In our patient, as well as in his, no systemic or ocular disorders were present to explain the lipid deposition in the cornea. Our case 1 is similar to a recent report of progressive bilateral lipid keratopathy by Castro-Rebollo et al. [11], where no neovascularization was observed, and the case was called primary.

Our patient 2 had a history of red eyes, pain, and photophobia showing on examination low-to-moderate inflammation at the corneo-scleral limbus (limbitis). This could imply a localized limbic vasculitis as a possible cause for the lipid deposition in the cornea with secondary peripheral corneal vascularization. In this case, in spite of the symptomatic control of the inflammation with topical steroids, the infiltration process progressed.

To our knowledge, this is the first paper to document the progression of this disorder and report confocal microscopy findings. These two cases were observed for more than a decade with documented slow and insidious progression of the infiltrates despite the efforts in diagnosis and treatment. In the vast majority of other reported cases, a penetrating keratoplasty was made necessary [8–10]. Differently, we showed that the visual acuity can remain quite good for years with very slow deterioration.

Confocal microscopy showed a pattern that resembled crystalline structures in the whole thickness of the stroma. These findings agree with early histopathological descriptions of the presence of cholesterol crystals deposited in the stroma [1, 8].

The nature of the lipid infiltration in primary and idiopathic cases is unclear, but we believe that as well as in our two cases, other idiopathic cases may have shown, at least in some instance, low-grade ocular inflammation, sometimes unnoticed by the patient. Our patient 1 had a history of red eyes as well, but she was not examined during that period.

The pathogenesis remains unclear, but we think that corneo-scleral microvascular changes with incompetent limbus blood vessels may have accounted for corneal lipid infiltration in these two cases. The small number of cases published with a histopathological study usually show the presence of vascularization in the corneal stroma, even though this neovascularization may not be evident in the slit lamp [11, 12]. Unfortunately, weak topical steroids seem not to completely control this inflammatory process, what could possibly stabilize and prevent the progression of the lipid infiltration.
